# An asymptomatic geminivirus activates autophagy and enhances plant defenses against diverse pathogens

**DOI:** 10.1007/s44154-024-00176-8

**Published:** 2024-10-08

**Authors:** Li Wang, Zijie Yu, Mengge Jiang, Mengyuan Tian, Hongsheng Zhou, Wanying Zhao, Ida Bagus Andika, Qiaoxia Shang, Liying Sun

**Affiliations:** 1 State Key Laboratory of Crop Stress Resistance and High-Efficiency Production, College of Plant Protection, Northwest A&F University, Yangling, 712100 China; 2 College of Plant Health and Medicine, Qingdao Agricultural University, Qingdao, 266109 China; 3 College of Bioscience and Resource Environment, Key Laboratory for Northern Urban Agriculture of Ministry of Agriculture and Rural Affairs, Beijing University of Agriculture, Beijing, 102206 China

**Keywords:** Apple geminivirus, Autophagy, Cross-protection, Pathogens, Resistance

## Abstract

Plant viral diseases cause great losses in agricultural production. Virus cross-protection is a strategy in which a mild virus is employed to shield plants against subsequent infections by severe viral strains. However, this approach is restricted to protection against the same viruses. In this study, we observed that pre-inoculation with apple geminivirus (AGV) reduced the accumulation of secondarily infected heterologous viruses, such as cucumber mosaic virus, potato virus X, and tobacco mosaic virus in *Nicotiana benthamiana*, tomato, and pepper plants. Transcriptional expression analysis showed that autophagy-related genes were transcriptionally up-regulated upon AGV inoculation at an early stage of infection. Accordingly, autophagic activity was observed to be elevated following AGV infection. Interestingly, AGV accumulation was reduced in autophagy-deficient plants, suggesting that autophagy activation promotes AGV infection in the plant. Moreover, pre-inoculation with AGV provided cross-protection against infection with a phytopathogenic bacterium (*Pseudomonas syringae*) and fungus (*Botrytis cinerea*) in *Nicotiana* species. In summary, our study showed that AGV, an asymptomatic virus, could protect plants against severe viral, fungal, and bacterial diseases to some extent through the activation of autophagy pathways, highlighting its potential as a biocontrol agent for managing a wide range of plant crop diseases in the field.

**Supplementary Information:**

The online version contains supplementary material available at 10.1007/s44154-024-00176-8.

## Introduction

Plant viral diseases are considered a major threat to agricultural production. Unlike other pathogens such as fungi and bacteria, viruses cannot be effectively controlled by chemical drugs. Therefore, the management of viral disease relies on preventing viruses from invading plants or employing plants resistant to viral infection. For decades, various techniques have been applied to generate virus-resistant plants, including conventional plant breeding and genetic engineering. Breeding resistant plants may be challenging when the required resistance genes are absent in genetically compatible wild relatives. Although modern genetic engineering technologies promise to solve plant viral disease-related issues, their effectiveness has not always been proven in the field (Gonsalves 2006; Zhao et al. 2020).

Virus cross-protection is a strategy to protect crop plants by using infections with mild viral strains to protect against secondary infections with more severe strains of the same viruses (Ziebell and Carr 2010). There are many examples of successful cross-protection in crops, particularly in commercial horticultural crops, including the use of an attenuated potato virus X (PVX) strain to protect tobacco and datura plants against challenge with a more severe strain of PVX (Salaman 1933). Another notable example is the use of this approach in citrus plants in South America, which provided protection against citrus tristeza virus (CTV). In field conditions, tomato crops have been protected against tobacco mosaic virus (TMV) through cross-protection (Rast 1972). To date, various mechanisms of cross-protection have been explored, including pathogen-derived resistance, RNA silencing, and exclusion/spatial separation. Among these mechanisms, RNA silencing is the primary driver of the cross-protection phenomenon. When a plant is initially infected with a mild strain of a virus, the RNA silencing machinery targets and degrades the viral RNA. If subsequently exposed to a more aggressive strain of the same virus, the existing RNA silencing response recognizes the similarities in genetic sequence between the strains, providing protection by degrading the RNA of the severe strain. However, the complexity of virus mixed infection suggests that it lacks a single, straightforward explanation. The molecular mechanisms and limitations affecting virus cross-protection remain largely unknown (Pechinger et al. 2019; Ziebell and Carr 2010). In addition to RNA silencing, autophagy is also thought to be pivotal in combating virus infection. Autophagy is a part of plant defense responses and serves as a fundamental mechanism against viruses (Choi et al. 2018; Ismayil et al. 2020b).

Autophagy is an evolutionarily conserved vacuole/lysosome-mediated process for degrading or recycling cellular contents/components such as cytosolic materials, macromolecules, and dysfunctional organelles in eukaryotic organisms (Liu and Bassham 2012; Yang and Bassham 2015). Three types of autophagy have been described in plants: microautophagy, macroautophagy, and mega-autophagy (Marshall and Vierstra 2018), of which macroautophagy (hereafter referred to as autophagy) is the major form (Mizushima 2007). Moreover, autophagy can be divided into selective autophagy and nonselective bulk autophagy depending on cargo specificity (Marshall and Vierstra 2018). The mechanism of autophagy is highly conserved among yeast, plants, and animals, with the core processes initiated by a series of autophagy-related (ATG) proteins (Lamb et al. 2013). The main machineries of autophagy can be classified into three groups: the core, consisting of ubiquitin-like complexes responsible for the formation of ATG8-PE; the E1-like enzyme ATG7; the E2-like enzyme ATG3; and the E3-like ATG5-ATG12-ATG16 conjugation systems. The ATG1/ATG13 kinase complex is required for autophagy induction, while the PI3K (phosphoinositide 3-kinase) complex involves ATG6/Beclin1, vacuolar protein sorting 15 (VPS15), VPS34, and ATG14 (Soto-Burgos et al. 2018; Xie and Klionsky 2007).

Research has documented that reducing or eliminating ATG genes, such as *ATG5* or *ATG7*, results in increased accumulation of a wide range of viruses, including both RNA viruses and DNA viruses (Hafren et al. 2017; Haxim et al. 2017; Jiang et al. 2019; Liu et al. 2005; Shukla et al. 2022; Yang et al. 2018). Conversely, some viruses exploit autophagic processes to facilitate their replication and movement (Huang et al. 2019; Li et al. 2017). Notably, autophagy commonly triggered by diverse abiotic and biotic stresses (Liu and Bassham 2012; Marshall and Vierstra 2018). Studies have also shown that several plant viruses or bacteria or fungi can induce autophagy (Haxim et al. 2017; Ismayil et al. 2020a; Li et al. 2018; Zhao et al. 2023). Several studies have demonstrated that autophagy plays a crucial role in plant immunity against various invading pathogens, such as bacteria, fungi, and viruses (Wang et al. 2014; Yang et al. 2020).

Apple geminivirus 1 (AGV) is a circular, single-stranded DNA virus (genus *Maldovirus*, family *Geminiviridae*) identified from apple trees in China (Liang et al. 2015). Notably, AGV has the capability to infect various herbaceous or horticultural plants without causing disease symptoms (Liang et al. 2015). In this study, we made the intriguing discovery that the pre-inoculation of AGV could reduce the accumulation of secondarily inoculated heterologous viruses, as well as other plant pathogens. Furthermore, analysis of key factors involved in autophagy revealed that AGV activates and exploits autophagy for its own accumulation. Additionally, we established a convenient inoculation method for AGV using *Agrobacterium*. Overall, this study describes an asymptomatic virus species that potentially confers cross-protection against diverse plant pathogens and further deepens our understanding of the mechanisms underlying cross-protection.

## Results

### Pre-inoculation with AGV reduces the accumulation of CMV, PVX, and TMV

As AGV was shown to be asymptomatic in some solanaceous plants (Liang et al. 2015), we were interested in whether AGV provides cross-protection against heterologous viruses. An infectious AGV DNA clone was inoculated using the agroinfiltration method as previously described (Liang et al. 2015). The infection of AGV was assessed by PCR (polymerase chain reaction) 7, 14, and 21 days post-inoculation (dpi). The assay showed AGV infection in inoculated leaves at 7 dpi, while no infection was detectable in the systemic upward leaves. At 14 and 21 dpi, AGV infection was detected in systemic leaves, indicating stable AGV replication in *N. benthamiana* plants (Supplementary Fig. 1A).

**Fig. 1:**
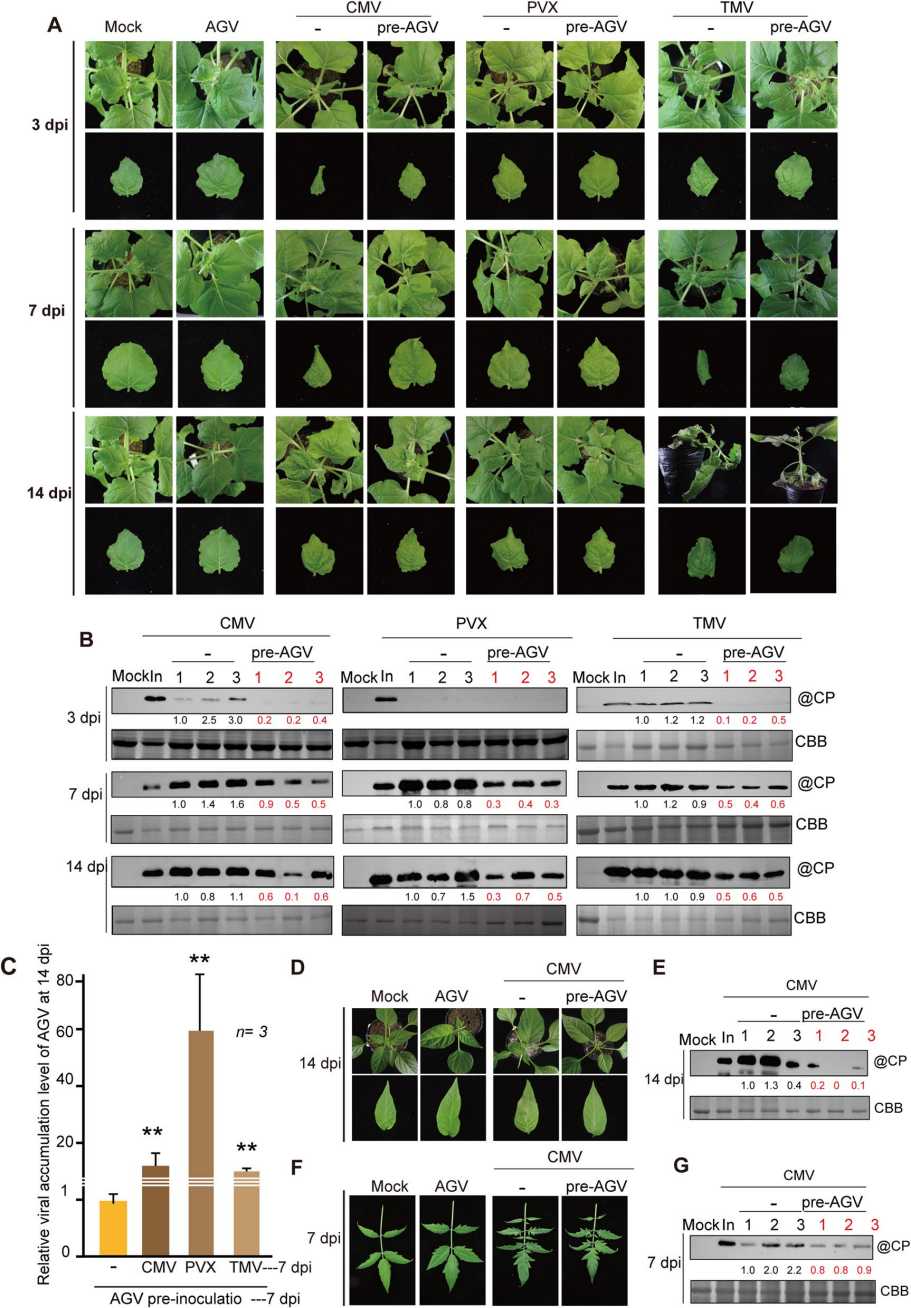
Effects of AGV pre-inoculation on the accumulation of secondarily inoculated heterologous viruses. **A**. Viral symptom expression in *N. benthamiana* plants pre-inoculated with AGV and inoculated with CMV, PVV, or TMV 7 days later. Plants were photographed 3, 7, and 14 days post-inoculation (dpi) of secondarily inoculated viruses. **B**. Western blot analysis of viral CP accumulation in the inoculated (In) and upper systemic leaves of the plants in the virus inoculation experiment described in **A**. Coomassie brilliant blue (CBB) staining was used as the loading control. The numbers below the blots (**B**, **E**, **G**) represent the relative signal levels quantified using ImageJ software (National Institutes of Health). The numbers were obtained after normalization with the signal levels of loading controls. **C**. Quantitative PCR analysis of the relative accumulation levels of AGV DNA in the upper systemic leaves of the plants described in **A**. **D** and **F**. Viral symptom expression in pepper plants (**D**) and tomato plants (**F**) pre-inoculated with AGV and inoculated with CMV 7 days later. The AGV singly infected sample was set to a value of 1.0. Vertical lines on the bars represent the SD. “**” indicates a significant difference at *P* < 0.01 (Student’s *t*‐test). **E** and **G**. Western blot analysis of viral CP accumulation in the inoculated (In) and upper systemic leaves of the pepper plants (**E**) and tomato plants (**G**) described in **D** and** F**

To investigate the possibility of AGV conferring cross-protection against other viral infections, we pre-inoculated AGV onto 4-week-old *N. benthamiana* plants and secondarily inoculated the plants 7 days later with the RNA viruses cucumber mosaic virus (CMV), PVX, and TMV using the mechanical inoculation method (Supplementary Fig. 1B). The viral symptoms and accumulation were assessed at 3, 7, and 14 dpi (Fig. 1A). Plants that were pre-inoculated with AGV but not challenged with a second virus, as well as plants that were only inoculated with the challenge viruses, were used as controls. Upper systemic leaves from all inoculated plants were collected for total protein and nucleic acid extraction. Interestingly, the plants pre-inoculated with AGV exhibited milder symptoms of CMV, PVX, and TMV infection than those only inoculated with the challenge viruses (Fig. 1A). The accumulation levels of CMV, PVX, and TMV were assessed by western blot analysis using antibodies specific for viral coat protein (CP). The analysis revealed that at 3, 7, and 14 dpi, a significant reduction occurred in viral CP accumulation in AGV pre-inoculated plants compared to plants that were only inoculated with the challenge viruses (Fig. 1B). These observations suggest that AGV has the capability to confer cross-protection against CMV, PVX, and TMV infections in *N. benthamiana*.

On the other hand, it is of interest to assess the effects of challenge viruses on AGV accumulation. Therefore, we quantified the relative level of AGV DNA accumulation using real-time quantitative PCR (qPCR). Total DNA was collected from the upper systemic leaves of the inoculated plants described above 7 days after inoculating CMV, PVX, and TMV. The assay showed a significant increase in AGV accumulation in the presence of CMV, PVX, and TMV compared to only AGV infection (Fig. 1C). These observations show that the secondary viral infection has a synergistic effect that benefits AGV accumulation, whereas AGV infection antagonizes the accumulation of the challenge viruses. This dynamic interplay between viral species provides valuable insights into the intricate interactions occurring during mixed viral infections.

To further investigate whether AGV induces cross-protection against severe viral infection in crop plants, we conducted experiments on economically important horticultural plants, namely tomato (*Solanum lycopersicum*) and pepper (*Capsicum annuum*). CMV is known to infect a wide array of horticultural crops, including pepper and tomato, making it one of the most detrimental viruses to these crops (Gallitelli 2000). Like the inoculation on *N. benthamiana*, AGV was pre-inoculated using *Agrobacterium*-mediated methods. After 7 dpi, the challenge virus, CMV, was inoculated using mechanical inoculation. Total proteins were extracted from the upper systemic leaves of tomato plants at 14 dpi (Fig. 1D) and from pepper plants at 7 dpi (Fig. 1F). The virus accumulation levels were assessed by western blot analysis using an antibody specific for CMV CP (Fig. 1E and 1G). As expected, the pre-inoculation of AGV resulted in milder CMV symptoms in tomato and pepper plants. The accumulation level of CMV CP was significantly reduced in AGV-pre-inoculated plants compared to those only inoculated with CMV. These results demonstrate that AGV could serve as an effective agent for cross-protection against severe viral infection in economically important horticultural plants.

### AGV infection up-regulates the transcription of genes involved in antiviral responses in *N. benthamiana*

To understand the mechanisms underlying AGV-induced cross-protection against heterologous viruses, we evaluated the expression levels of key genes involved in various antiviral pathways at different stages of AGV infection in *N. benthamiana* plants. Viral infection was confirmed by PCR using AGV-specific primers (Supplementary Fig. 1A, Supplementary Table 1). The signaling pathways of salicylic acid (SA), jasmonic acid (JA), and ethylene (ETH) have been reported to play roles in plant defense responses (Bari and Jones 2009; Singh et al. 2004; Yan and Xie 2015). The transcript expression levels of pathogenesis-related protein (PR)1 and PR2 genes often serve as markers for SA signaling and are related to systemic acquired resistance (SAR). Mitogen-activated protein kinase 3 (MPK3) and Myelocytomatosis transcription factor 2 (MYC2) are key regulators of JA signaling. Ethylene response factor 3 (ERF3) serves as a marker gene for monitoring ethylene signal transduction (Pitzschke et al. 2009; Qi et al. 2018; Velivelli et al. 2015; Yan and Xie 2015). We analyzed the transcript expression levels of PR1, PR2, MPK3, MYC2, and ERF3 to represent the responses mediated by SA, JA, and ETH signaling pathways. Furthermore, studies have demonstrated that RNA interference (RNAi) is a highly conserved antiviral immune mechanism that plays a vital role in plant defense against viral infection (Calil and Fontes 2017; Yang and Li 2018). The expression of core components of the RNAi pathway, such as Dicer-2 (DCL2), Dicer-4 (DCL4), and Argonaute-2 (AGO2), has been reported to be induced by viral infection (Bai et al. 2012; Deleris et al. 2006). Therefore, we assessed the expression of *DCL2*, *DCL4*, and *AGO2* to monitor RNAi activity in *N. benthamiana* following AGV inoculation. Geminivirus infection has been reported to enhance autophagy (Haxim et al. 2017). To examine whether AGV infection stimulates autophagic activity, we analyzed the transcript expression of ATG genes in *N. benthamiana* plants with or without AGV infection.

**Fig. 2:**
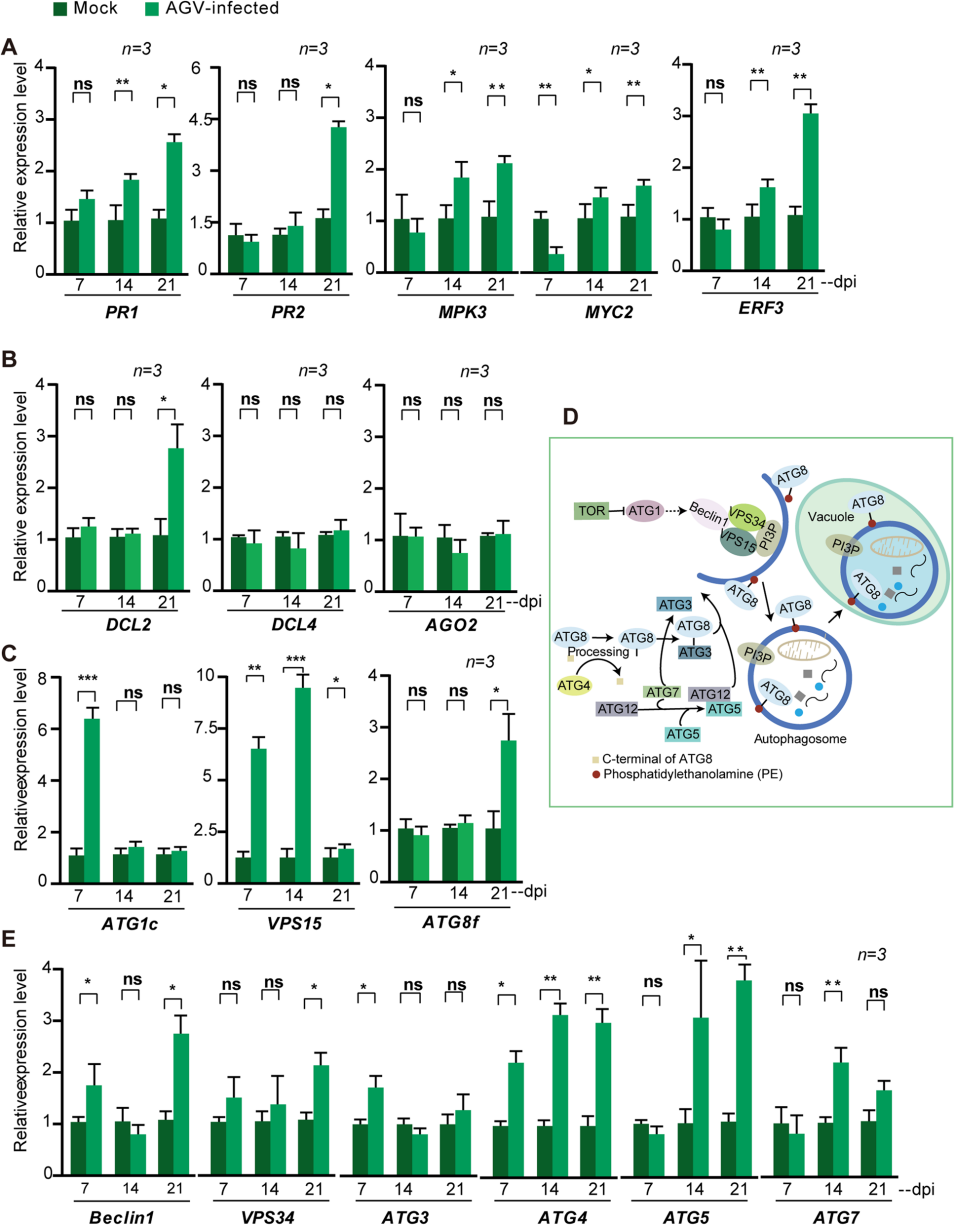
Effects of AGV infection on the transcriptional expression levels of genes associated with antiviral defenses in *N. benthamiana* plants, analyzed by quantitative real-time RT-PCR. **A**. Relative accumulation levels of the transcripts of genes associated with salicylic acid signaling (*PR1* and *PR2*), jasmonic acid signaling (*MPK3* and *MYC2*). **B**. Relative accumulation levels of the transcripts of genes associated with RNA silencing (*DCL2*, *DCL4* and *AGO2*). The mock-inoculated sample was set to a value of 1.0. Vertical lines on the bars represent the SD. “*” and “**” indicate a significant difference at *P* < 0.05 and 0.01, respectively (Student’s *t-*test). **C**. An illustration depicting autophagy pathways in the plant cell. **D** and** E**. Relative accumulation levels of the transcripts of genes associated with autophagy pathways in the plants

As shown in Fig. 2, the transcription levels of key genes in the SA pathway, *PR1* and *PR2*, were up-regulated at 14 and 21 dpi upon AGV infection compared to the control (mock) plants. Similarly, the transcription levels of key genes related to the JA pathway, *MPK3* and *MYC2*, as well as the key ETH gene, *ERF3*, showed a slight up-regulation at 14 dpi. Additionally, the transcription levels of the *DCL2*, *DCL4*, and *AGO2*genes, involved in the antiviral RNAi pathway, only *DCL2* exhibited up-regulation at 21 dpi (Fig. 2A). These results suggest that AGV infection induces hormone signaling and RNAi-mediated plant defense at 14 dpi. Interestingly, the expression levels of ATGs were found to be consistently up-regulated at all stages of AGV infection (Fig. 2D and E). Notably, ATGs involved in the initiation of autophagy, such as ATG1, VPS15, ATG3, and Becline1/ATG6 (Fig. 2C), showed significant up-regulation at 7 dpi, an early stage of AGV infection (Fig. 2D and E), even when AGV accumulation was still low (Supplementary Fig. 1B). Moreover, the expression of ATGs associated with ATG8-PE (lipid phosphatidylethanolamine (PE) conjugated ATG8) formation, including ATG4, ATG5, ATG7 and ATG8f was predominantly elevated at a later stage of AGV infection (14 and 21 dpi; Fig. 2D and E), consistent with the observation that pre-inoculation with AGV readily provided protection against severe virus infection before 10 dpi, considered an early stage of virus inoculation (Fig. 1B). These observations suggest that pre-inoculation with AGV triggers an antiviral mechanism during the early stage of AGV infection. Moreover, the results indicate that ATGs are readily induced at an early stage of AGV infection, suggesting the potential role of autophagy as a major contributing factor in AGV-mediated cross-protection.

### Mutual promotion between AGV infection and autophagy activity

**Fig. 3:**
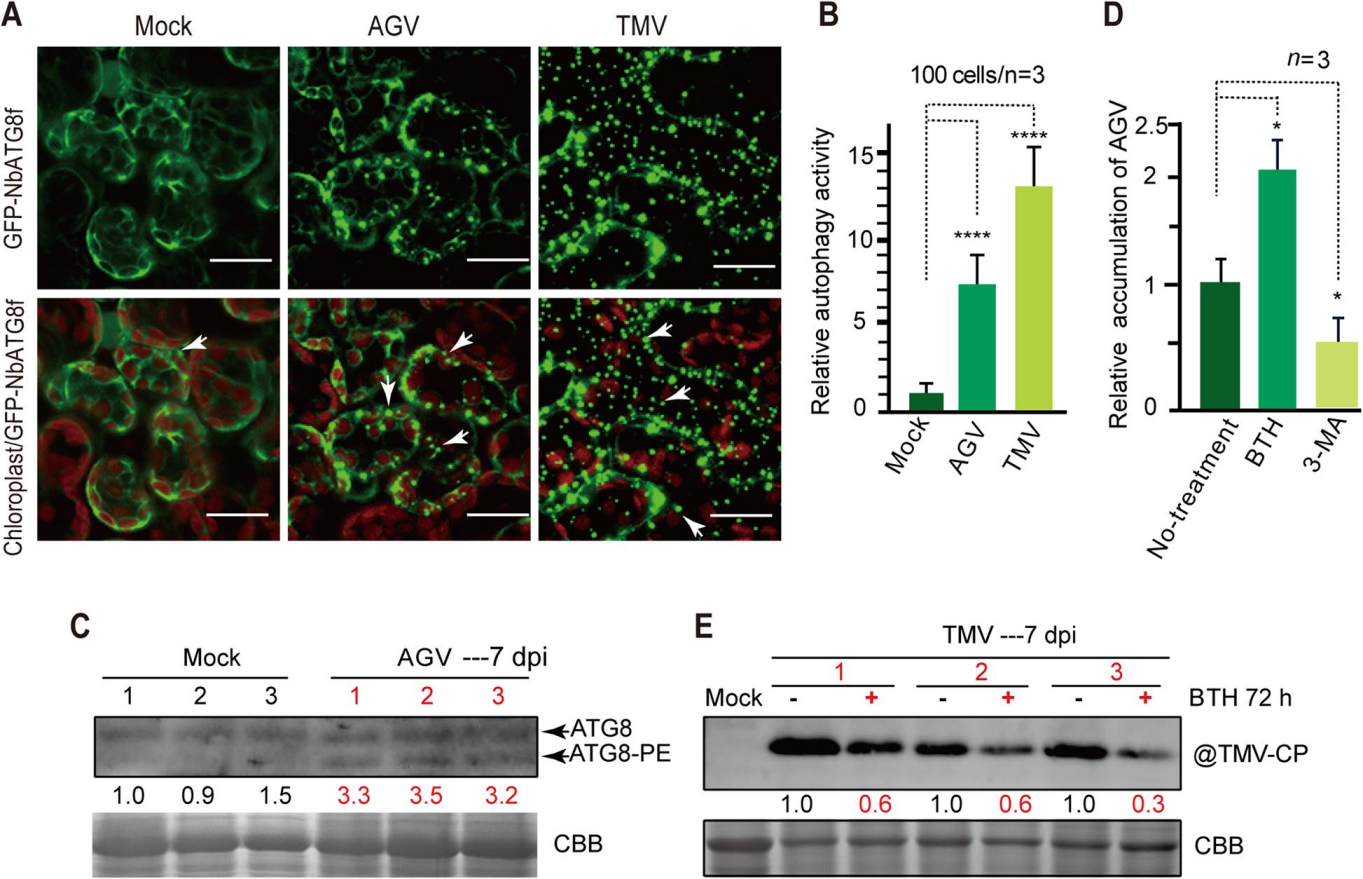
Induction of autophagy by AGV infection. **A**. Effect of AGV or TMV infection on autophagic activity assessed by using an *N. benthamiana* autophagy marker (NbATG8f) fused with GFP (GFP‐NbATG8f). *Agrobacterium* cultures carrying the GFP‐NbATG8f binary vector were used to infiltrate the leaves of AGV- or TMV- infected plants, and 2 days after infiltration, leaves were sampled and the GFP fluorescence in epidermal cells was observed by confocal laser scanning microscopy. Scale bars, 20 μm. **B**. Quantification of autophagic activity based on the numbers of GFP‐NbATG8f‐labeled autophagic structures in the cells of leaves described in **A**. The mock-inoculated sample was set to a value of 1.0. Vertical lines on the bars represent the SD. “***” indicates a significant difference at *P* < 0.001 (Student’s *t-*test). **C**. Accumulation of ATG8 and its lipidated form (ATG8‐PE) in the leaf tissue of plants infected with AGV. Total protein samples were extracted from the leaves and subjected to immunoblotting analysis using anti-ATG8 antibody. Coomassie brilliant blue (CBB) staining was used as the loading control. The numbers below the blots (**C**, **E**) represent the relative signal levels quantified using ImageJ software (National Institutes of Health). The numbers were obtained after normalization with the signal levels of loading controls. **D**. Quantitative PCR analysis of the relative accumulation levels of AGV DNA in upper systemic leaves treated with an autophagy inducer, benzothiadiazole (BTH) and an autophagy inhibitor, 3-methyladenine (3-MA). The non-treated sample was set to a value of 1.0. Vertical lines on the bars represent the SD. “*” indicates a significant difference at *P* < 0.05 (Student’s *t-*test). **E**. Western blot analysis of TMV CP accumulation in upper systemic leaves treated with BTH

To further investigate the role of AGV infection in stimulating autophagic activity and activating plant defenses, we compared the levels of autophagic activity between plants inoculated with AGV and uninoculated plants. ATG8 commonly serves as a marker for monitoring autophagic activity (Bassham 2015). In this study, we utilized a green fluorescence protein (GFP) tagged to the N-terminal of ATG8f (GFP-NbATG8f) as a marker to represent autophagosomes (Contento et al. 2005). We transiently expressed GFP-NbATG8f by agroinfiltration in leaf tissues and observed the GFP-labeled autophagic bodies by using confocal laser scanning microscopy (Fig. 3A). As a previous study reported that TMV infection increases autophagic activity in *N. benthamiana* (Liu et al. 2005), we included TMV infection in this experiment. Like TMV infection, AGV infection significantly increased the punctate fluorescent signals of GFP-NbATG8f compared to those in mock plants (Fig. 3A white arrows). Quantification of the GFP-labelled punctates (Fig. 3A) revealed that the number of autophagic bodies was significantly increased in AGV-infected (approximately 7.0-fold) and TMV-infected (approximately 14-fold) plants (Fig. 3B). Autophagosome formation requires the activation of ATG8 by ATG4 and the conjugation of activated ATG8 to phosphatidylethanolamine (PE) lipids. The accumulation level of lipidated ATG8, also referred to as ATG8-PE, reflects the level of autophagic activity in the cell (Bassham 2015). We carried out SDS-PAGE in the presence of urea to separate ATG8-PE from ATG8 (Niu et al. 2022). Immunoblotting usually detects ATG8 and ATG8-PE as slower- and faster-migrating bands. As shown in Fig. 3C, the accumulation of ATG8-PE was elevated upon AGV infection compared to that in mock plants. These results indicate that autophagy is activated following AGV infection.

To investigate the effect of autophagic activation on AGV infection, we utilized an autophagy activator, benzothiadiazole (BTH), a functional analog of SA, and an autophagy inhibitor, 3-methyladenine (3-MA), which is commonly used to modulate autophagic activity (Niu et al. 2022; Takatsuka et al. 2004). *N. benthamiana* plants infected with AGV or TMV were treated with BTH and 3-MA using a half-leaf approach, as previously described (Huang et al. 2019; Niu et al. 2022). As shown in Fig. 3, the accumulation level of AGV genomic DNA was significantly increased upon treatment with BTH, while it decreased following treatment with 3-MA, as compared to the virus accumulation in the untreated leaf tissues (Fig. 3D). These results suggest that autophagy benefits AGV accumulation. In contrast, TMV accumulation was suppressed with BTH treatment (Fig. 3E), indicating that autophagy restricts TMV infection. Taken together, these data propose that AGV activates autophagy to promote its own replication, but this activation suppresses infection of secondarily challenged viruses.

**Fig. 4:**
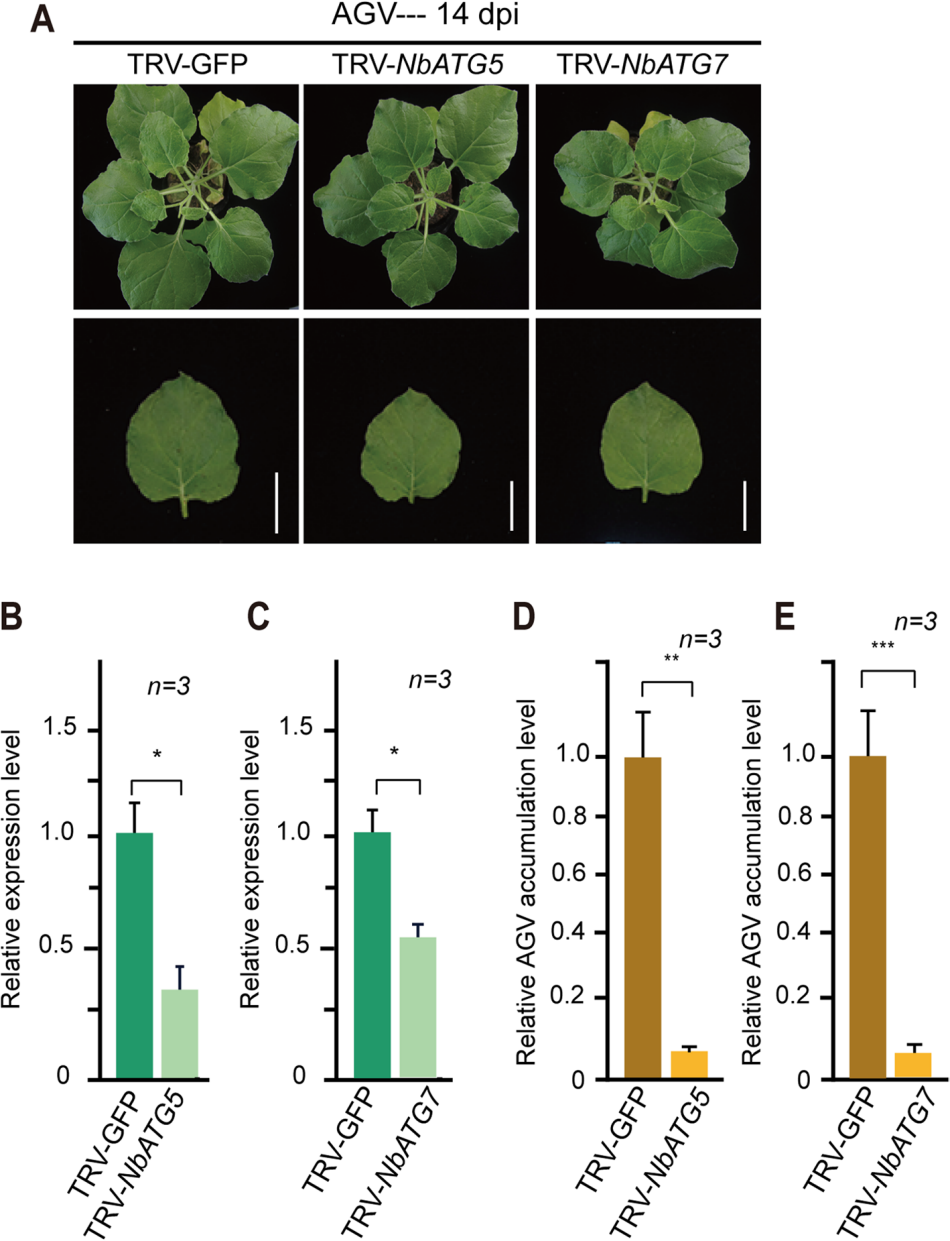
AGV DNA accumulation in autophagy-deficient plants. **A**. AGV infection in plants with *NbATG5* and *NbATG7* genes silenced using TRV-VIGS. Plants were inoculated with TRV–*NbATG5* and TRV-*NbATG7* or TRV-GFP as a control, and 10 days later, plants were inoculated with AGV. Lower panels are the images of the upper leaf. Scale bars, 1 cm. **B** and** C**. Relative accumulation levels of the transcripts of *NbATG5* and *NbATG7* genes in the plants described in **A**. The TRV-GFP-infected sample was set to a value of 1.0. Vertical lines on the bars represent the SD. “*” indicates a significant difference at *P* < 0.05 (Student’s *t-*test). **E** and** F**. Quantitative PCR analysis of the relative accumulation levels of AGV DNA in the upper systemic leaves of the plants described in **A**. The TRV-GFP-infected sample was set to a value of 1.0. Vertical lines on the bars represent the SD. “*,” “**,” and “***” indicate a significant difference at *P* < 0.05, < 0.01, and < 0.001, respectively (Student’s *t*‐test)

To further confirm this view, we next examined AGV replication in autophagy-deficient host plants. We silenced the ATG5 and ATG7 genes of *N. benthamiana* (*NbATG5* and *NbATG7*) using TRV-VIGS as previously reported (Niu et al. 2021). The levels of *NbATG5* or *NbATG7* mRNA were significantly reduced in TRV-*NbATG5*- or TRV-*NbATG7*-infected plants at 14 dpi compared to TRV-GFP-infected plants, used as non-silenced control plants (Fig. 4B, C). Subsequently, we inoculated the plants with the AGV infectious clone through an *Agrobacterium*-mediated delivery system. There were no observable AGV symptoms in the *NbATG5*- and *NbATG7*-silenced plants, as well as in non-silenced plants (Fig. 4A). The AGV accumulation in upper systemic leaves was assessed by qPCR. As expected, the AGV accumulation was significantly reduced in *NbATG5*- and *NbATG7*-silenced plants compared to that in control plants (Fig. 4D, E). These data collectively indicate that autophagy promotes AGV replication.

### Pre-inoculation with AGV reduces the growth of *Pseudomonas syringae* and *Botrytis cinerea* in *Nicotiana* species plants

**Fig. 5:**
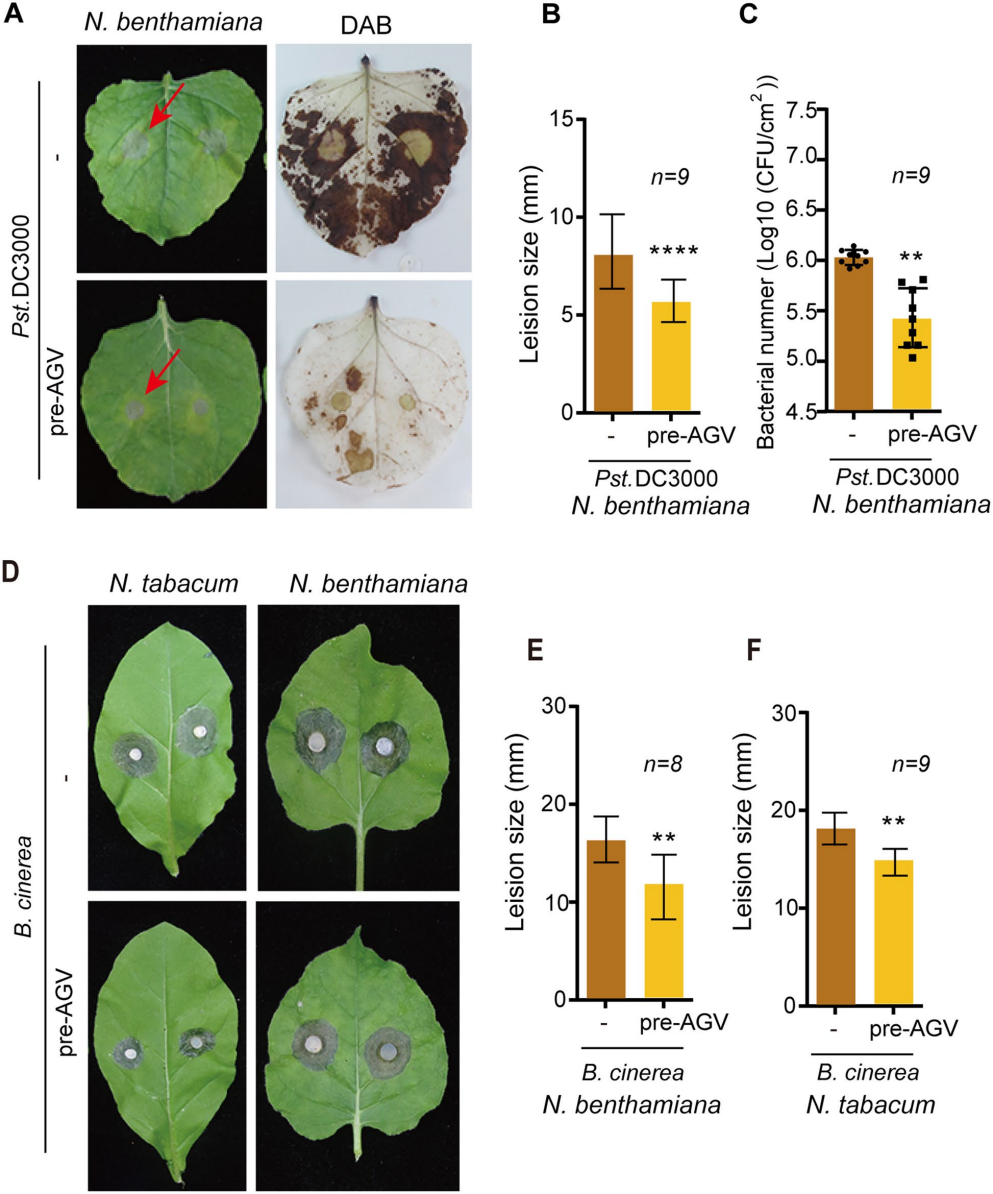
Effects of pre-inoculation with AGV on bacterial and fungal infections. **A** and **D**. Development of lesions in AGV-infected leaves inoculated with *P. syringae* (*Pst* DC3000) (**A**) and *B. cinerea* (**D**). Plants were pre-inoculated with AGV and, 21 days later, were inoculated with *P. syringae* and *B. cinerea*. Plants were photographed at 4 and 3 days after bacterial and fungal inoculation, respectively. DAB staining was also performed after *Pst* DC3000 infection. **B**, **E** and **F**. Bacterial (**B**) and fungal (**E**,** F**) lesion areas measured on the leaves described in **A** and **D**. **C**. Bacterial population of *Pst* DC3000 at 2 days after inoculation at leaves with or without pre-inoculation of AGV**.** Vertical lines on the bars represent the SD. “**” and “****” indicates a significant difference at *P* < 0.01 and 0.0001, respectively (Student’s *t-*test)

Given that AGV infection activates autophagy and other defense pathways, we further investigated whether AGV infection can confer protection against bacterial or fungal pathogens. Autophagy has been reported to play a pivotal role in plant defense against the necrotrophic fungal pathogen *Botrytis cinerea*. Disruption of key autophagy-related genes, such as ATG5, is shown to result in more severe necrosis symptoms, heightened infection, and increased reactive oxygen species (ROS) activity in *Arabidopsis thaliana* (Wang et al. 2014). On the other hand, autophagy is also involved in *Pseudomonas syringae* infection. The deletion of *AtATG5* led to reduced ROS accumulation and increased growth rate of *P. syringae* strains (Wang et al. 2014).To evaluate the effects of plant immunity triggered by AGV infection against bacterial or fungal pathogens, we compared the pathogenicity of the virulent *P. syringae* pv. tomato DC3000 (*Pst* DC3000) and *B. cinerea* between *N. benthamiana* plants with or without pre-inoculation with AGV. The results showed that AGV infection limited disease progression. Both *Pst* DC3000 and *B. cinerea* produced smaller lesions on the leaves of plants with AGV pre-inoculation compared to those on AGV-free plants (Fig. 5A, B, D, E, F). Moreover, leaves pre-inoculated with AGV show reduced ROS activity and lower *P. syringae* population during *Pst* DC3000 infection, compared to AGV-free (Fig. 5A, C). These results imply that pre-inoculation of AGV can inhibit *Pst* DC3000 infectivity, leading to diminished ROS responses.

**Fig. 6:**
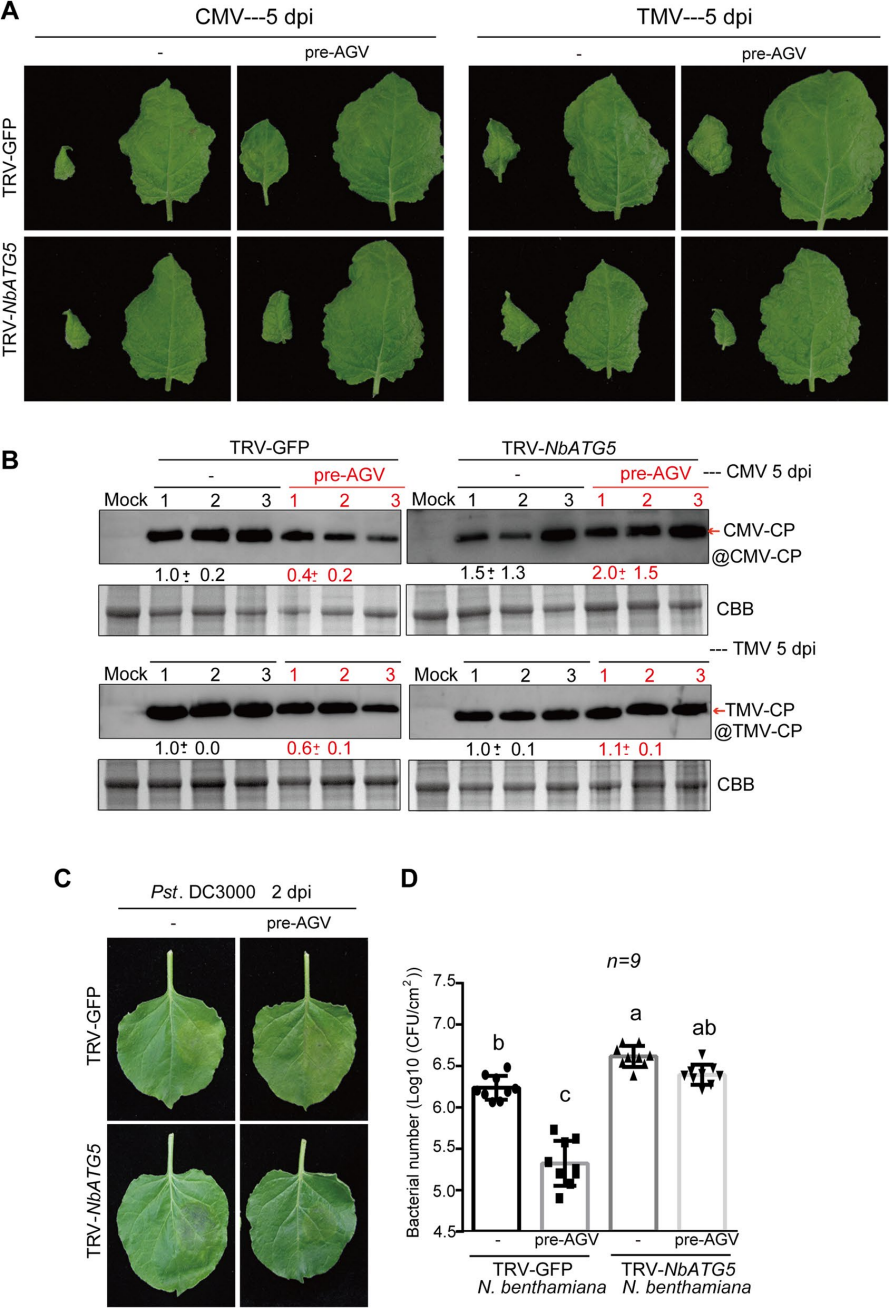
Impact of *NbATG5*-silencing on AGV-induced protection against viral and bacterial pathogens. **A**. Plants with TRV-*NbATG5* gene silenced by TRV-VIGS were pre-inoculated with or without AGV and subsequently challenged with CMV or TMV. The right panel displays plants inoculated with TRV-*NbATG5*, while the left panel serves as a control with TRV-GFP-inoculated plants. Following a 10 days inoculation period, AGV pre-inoculated was performed and maintained for 14 days. Subsequently, CMV and TMV were inoculated and upper systemic leaves were photographed at 5 dpi. **B**. Western blot analysis was conducted to assess CMV or TMV accumulation using specific antibodies against viral coat protein in the upper systemic leaves of plants described in A. Coomassie brilliant blue (CBB) staining served as the loading control. Values below the blots indicate quantified relative signal levels using ImageJ software (National Institutes of Health), normalized to loading controls levels. **C**. *P. syringae* (*Pst* DC3000) was inoculated in TRV and AGV-infected leaves as described in **A**. Leaves were photographed at 2 days post-bacterial inoculation. **D**. Bacterial population of *Pst* DC3000 in leaves of **C**. The vertical lines on the bars represent the standard deviation. The different letters show a significant difference at p < 0.05 (one-way ANOVA)

To investigate the protective effects of pre-inoculation with AGV against virus infection by promoting autophagy activity, we pre-inoculated *NbATG5* silenced plants with AGV (Fig. 4), and then challenged with CMV and TMV. As anticipated, control plants treated with TRV-GFP and pre-inoculated with AGV displayed milder CMV or TMV symptoms on both newly grown (left) and 5-day-old (right) upper systemic leaves compared to plants without AGV inoculated (Fig. 6A upper panel). However, plants in which *NbATG5* expression was suppressed by TRV-*NbATG5* showed no observable difference in symptoms between those pre-AGV and those without AGV (Fig. 6A below panel). The accumulation of the challenging viruses CMV or TMV in the upper systemic leaves was also evaluated through western blot analysis. As expected, CMV/TMV accumulation was significantly lower in pre-inoculated plants of the control group (TRV-GFP), but not in *NbATG5* -silenced plants (Fig. 6B). Following the same processes of challenging virus inoculation, the plants described in Fig. 6A were inoculated with *Pst* DC3000 as well (Fig. 6C). Consistent with the results shown in Fig. 6A and B, pre-inoculation with AGV provided protection against *Pst* DC3000 infection in control plants (TRV-GFP), resulting in milder disease symptoms and a significantly reduction in bacterial numbers. However, there was no observable difference in *NbATG5*-silenced plants (TRV-*NbATG5*) (Fig. 6C and D). These results suggest that pre-inoculation with AGV confers protection against viral, bacterial or fungal pathogens such as CMV/TMV, *Pst* DC3000 and *B. cinerea*, depending on authophagy activity.

### Establishment of an AGV inoculation method in various solanaceous plants

**Fig. 7:**
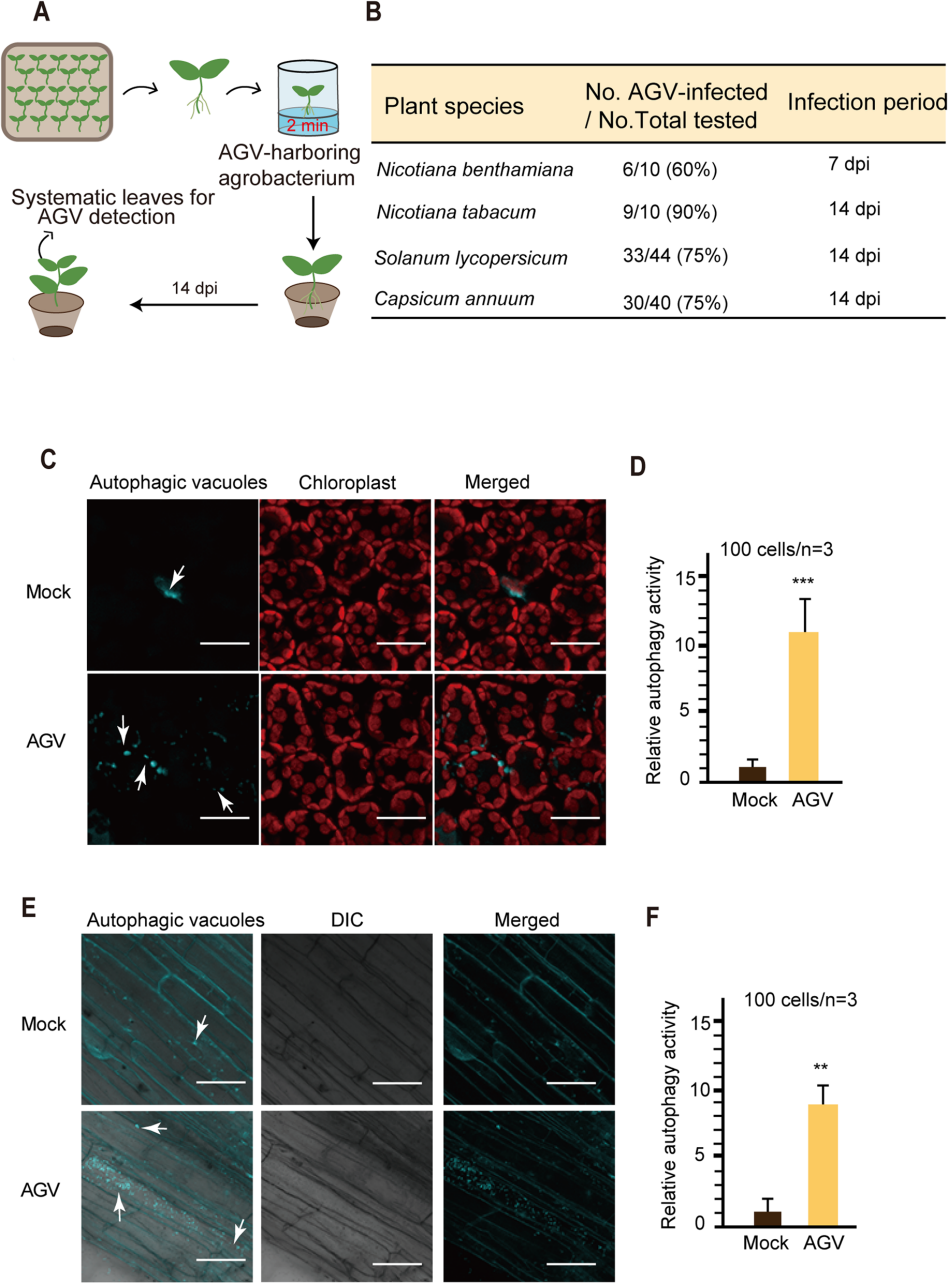
Establishment of an AGV inoculation method through *Agrobacterium*-mediated inoculation on root plants.** A**. Illustration describing the step-by-step procedure for inoculating AGV on the roots of various solanaceous plants using *Agrobacterium* cultures carrying an infectious cDNA clone of AGV. **B**. Efficiency of AGV infection through the inoculation method described in A. AGV DNA was detected in upper systemic leaves by PCR. **C** and **E**. Autophagic activity in leaf (**C**) and root (**E**) tissues of AGV-infected tomato revealed by visualizing autophagy vesicles through fluorescent dye monodansylcadaverine (MDC) staining and confocal laser scanning microscopy. Scale bars, 20 μm. **D** and **F**. Quantification of autophagic activity based on the numbers of MDC-stained autophagic structures in the cells of the leaf (**D**) and root (**F**) tissue described in **C** and **E**. The mock-inoculated sample was set to a value of 1.0. Vertical lines on the bars represent the SD. “**” and “***” indicates a significant difference at *P* < 0.01 and 0.001, respectively (Student’s *t-*test)

Many solanaceous crops, such as tobacco, tomato, and pepper, are commonly transplanted from nursery beds to the field for further growth and production. The pre-inoculation of AGV could provide cross-protection against severe symptom-inducing viruses such as CMV, PVX, and TMV (Fig. 1). Thus, we wanted to establish a simple and efficient method to inoculate AGV on young plants during transplantation to achieve cross-protection. Seedlings of tobacco, *N. benthamiana*, tomato, and pepper were grown in nursery pots. When the seedlings were transplanted into new pots, they were first removed from the nursery pot, and their roots were gently detached from the planting medium. The roots were then soaked in AGV infectious clone-harboring *Agrobacterium* liquid for 2 min prior to planting in a pot for further growth (Fig. 7A). AGV infection was detected in the upper systemic leaves using PCR. This inoculation method for AGV achieved a relatively high rate of infection (60 − 90%, Fig. 7B).

We examined the autophagic activity in the leaf and root tissues of AGV-infected tomato by monitoring autophagic vesicles through fluorescent dye monodansylcadaverine (MDC) staining (Contento et al. 2005). The number of stained autophagic bodies (blue color, white arrows) in the leaf (Fig. 7C and D) and root (Fig. 7E and F) tissues of tomato plants infected with AGV was greater than in those of AGV-free plants. These results demonstrate that autophagic activity is enhanced in tomato leaf or root tissues upon AGV infection through the *Agrobacterium*-mediated root inoculation method. The induction of autophagy in plants through this method suggests the potential of this AGV inoculation approach for controlling viral diseases of crop plants on a large agricultural scale.

## Discussion

Cross-protection, also known as cross-immunity, is an important strategy in the management of plant viral diseases (Pechinger et al. 2019; Ziebell and Carr 2010). In this study, we observed that a geminivirus, AGV, could provide protection against subsequent severe infection by heterologous viruses to some extent. When plants are pre-inoculated with AGV, plant defense responses are activated, making the plant more resistant to secondarily invading viruses (Fig. 1). Hence, AGV can be used as an attenuated virus in a cross-protection strategy for controlling severe diseases induced by heterologous virus. AGV thereby differs from the conventional virus, which only provides protection against secondary infection with more virulent strains of the same or closely related viruses. This phenomenon implies that the mechanism underlying AGV-triggered cross-protection is distinct from that of traditional cross-protection based on viral sequence specificity. In traditional cross-protection, when a plant is infected with a mild strain of a particular virus, it triggers the production of small RNA molecules known as small interfering RNAs (siRNAs). These siRNAs are then incorporated into an RNA-induced silencing complex (RISC), which targets and degrades viral RNA molecules with a complementary sequence to the siRNAs (Yang and Li 2018). During the RNA silencing process, siRNA can spread systemically throughout the plant, targeting, and degrading viral RNAs in uninfected cells and providing protection against subsequent infections with related viruses (Ratcliff et al. 1999; Stram and Kuzntzova 2006). This effective targeting and degradation of closely related viral sequences by siRNAs provides strong cross-protection. However, viruses with more divergent sequences may not be effectively targeted by existing siRNAs, limiting the extent of cross-protection.

Since DCLs and AGOs play crucial roles in RNAi responses, the transcription levels of these genes are commonly up-regulated during virus infection (Bai et al. 2012; Deleris et al. 2006). In this study, we selected the *DCL2*, *DCL4,* and *AGO2* genes for qRT-PCR analysis to monitor the activation of RNAi antiviral immunity. The transcript levels of these genes were not significantly up-regulated at the early stage of AGV infection, indicating that RNA-based antiviral immunity was not induced at the early stage of AGV infection (Fig. 2A). We also evaluated hormone-mediated plant immunity by monitoring the expression levels of marker genes involved in SA, JA, and ETH signaling transduction (Fig. 2A). The regulation profile of hormone signaling transduction by AGV was similar to RNAi responses, meaning that AGV did not trigger hormone-mediated immunity at the early stage of infection. Interestingly, when we examined autophagy-related immunity, we found that the transcript levels of several key genes involved in autophagy were drastically increased at the early stage of AGV infection. As AGV triggered cross-protection against severe viral infection at an early stage, the autophagy responses to AGV infection may provide an important clue to understanding the mechanism of this cross-protection against heterologous viruses. Interestingly, our data showed that pre-inoculation with AGV can decrease lesion development associated with bacterial or fungal infection (Fig. 6). Bacterial and fungal pathogens are known to be constrained by hormone-mediated and autophagic immune reactions (Denance et al. 2013; Lai et al. 2011; Ustun and Hofius 2018; Wang et al. 2014).

Autophagy is an evolutionarily conserved intracellular recycling process that maintains cellular homeostasis (Mizushima 2007). Autophagy could play dual roles in virus infection. On one hand, autophagy can function as an antiviral defense mechanism by targeting invading viruses (Hafren et al. 2017; Haxim et al. 2017; Jiang et al. 2019; Liu et al. 2005; Niu et al. 2022). On the other hand, certain viruses have evolved mechanisms to exploit autophagy for their own benefit, they manipulate the autophagy machinery to promote their own replication and survival within the host cell (Huang et al. 2019; Li et al. 2017). For example, these viruses can manipulate the autophagy process to form protective membranous structures that shield viral replication complexes from host immune responses or utilize autophagosomes for the transport of viral proteins to other cells (Huang et al. 2019; Li et al. 2017). Therefore, the role of autophagy in virus infection is complex and can vary depending on the specific viruses and hosts. In this study, we demonstrated that the activation of autophagy promoted AGV accumulation while suppressing TMV accumulation (Fig. 3). Thus, in the case of a mixed infection of AGV and challenge viruses, autophagy plays both antiviral and proviral roles, with the outcome determined by the interplay between the virus species and the host’s immune responses.

*Geminiviridae* is one of the largest virus families and currently contains 14 genera. All of the genera in *Geminivridae* have a single genomic component except for the genus *Begomovirus*, which contains viruses with either a single genomic component or two genomic components. Cotton leaf curl Multan virus (CLCuMuV), tomato yellow leaf curl virus (TYLCV), and tomato yellow leaf curl China virus (TYLCCNV) are members of the genus *Begomovirus*. CLCuMuV and TYLCCNV have two genomic components, while TYLCV only has one (Nawaz-ul-Rehman and Fauquet 2009). Unlike AGV, these geminiviruses induce severe disease symptoms in host plants, including the experimental plant *N. benthamiana* (Haxim et al. 2017; Jia et al. 2016). Like AGV, CLCuMuV, TYLCV, and TYLCCNV have been shown to activate autophagy in plants and the insect whitefly vector (Haxim et al. 2017; Luan et al. 2011; Wang et al. 2016). However, the activation of autophagy was shown to confer resistance to CLCuMuV, TYLCV, and TYLCCNV infection in plants and whiteflies (Haxim et al. 2017; Wang et al. 2016). In ATG5- or ATG7-silenced plants, the accumulation of CLCuMuV, TYLCV, and TYLCCNV was enhanced (Haxim et al. 2017). Viral proteins, commonly viral CP or RNA silencing suppressor, are targeted by autophagy. It has been shown that βC1, a silencing suppression protein encoded by CLCuMuV, interacted with NbATG8 and was subjected to autophagic degradation (Haxim et al. 2017). Disrupting the interaction of βC1 with ATG8 in the mutant virus, which encodes a mutated βC1, prevented βC degradation, resulted in more severe symptoms, and increased viral DNA accumulation (Haxim et al. 2017). These observations indicate that autophagy could play a varying role during virus infection, even among viruses from same family, in this case, *Geminiviridae*. Further studies are necessary to fully understand the mechanisms underlying the role of autophagy in virus infection and to explore the potency of regulating autophagy to control plant diseases caused by viruses and other pathogens.

## Conclusion

In this study, we observed that pre-inoculation with AGV reduced the accumulation of various secondarily infected heterologous viruses in tobacco, tomato, and pepper plants. Transcriptional expression analysis revealed the up-regulation of autophagy-related genes upon AGV inoculation, indicating an early activation of autophagy in response to the virus. This enhanced autophagic activity was triggered by AGV infection. Moreover, the activation of autophagy was shown to promote AGV accumulation. We also demonstrated that pre-inoculation with AGV provided cross-protection against a phytopathogenic bacterium (*P. syringae*) and fungus (*B. cinerea*) in *Nicotiana* species. Overall, our study suggests AGV has the potential to serve as a biocontrol agent for managing a diverse range of plant crop diseases, including severe viral, fungal, and bacterial infections, through the activation of autophagy pathways.

## Materials and methods

### Plants, viruses, bacterium, and fungus

*N. benthamiana*, *N. tabacum*, *S. lycopersicum,* and *C. annuum* plants were grown in soil and kept in a greenhouse at 25 ℃ with 16 h of daylight. Apple geminivirus 1 (AGV, KM386645) was isolated from apple plants in Yangling, Shaanxi province, China. An infectious AGV DNA clone (Yangling isolate) was described previously (Liang et al. 2015). CMV isolate Fny was obtained from Xianbin Wang (CAU, China Agricultural University) and accession number was reported previously (Zhao et al. 2022). TMV (AF165190), PVX (NC_011620.1), were isolated from potato plants (*Solanum tuberosum*) obtained from Yulin City, Shaanxi Province, China. *P. syringae* pv. *tomato* DC3000 and *B. cinerea* were generously provided by Qing Ma (Northwest A&F University).

### Virus inoculation and Agrobacterium infiltration

For virus mechanical inoculation, leaves of *N. benthamiana*, *N. tabacum*, *S. lycopersicum,* and *C. annuum* plants at the stage with 4–5 leaves were dusted with quartz sand powder and CMV, PVX, and TMV particles diluted in 10 mM sodium phosphate buffer (PB; pH 7.0) and rubbed as previously described (Hull 2009). *N. benthamiana* leaves with mosaic symptoms due to CMV, PVX, or TMV infection were stored at − 80 ℃ and used as viral inoculum for mechanical inoculation.

For the agroinfiltration of leaf tissue, *Agrobacterium* harboring the plasmid constructs was cultured in liquid Luria–Bertani medium with appropriate antibiotics at 28 ℃ overnight, collected by centrifugation at 12,000 rpm for 1 min, and resuspended in the infiltration buffer (10 mM MES pH 5.6, 10 mM MgCl_2_, 100 μM acetosyringone). The suspensions were diluted to OD_600_ = 0.8 and stored in the dark at room temperature for 3–4 h before infiltration.

For the *Agrobacterium*-mediated inoculation of AGV on root tissue, *N. benthamiana*, *N. tabacum*, *S. lycopersicum,* and *C. annuum* seedlings (two-leaf stage) were gently detached from the nursery pot and planting medium. The roots of the seedlings were dipped into the *Agrobacterium* (harboring the AGV infectious clone) infiltration solution described above for 2 min before transplantation into a new pot. The plants were grown in a greenhouse under a 16-h light/8-h dark cycle at 25 °C. The systemic upper leaves were sampled for the detection of AGV at 14 dpi.

### RNA extraction and RT-qPCR

Total RNA was extracted from virus-infected leaves collected from at least three plants using Trizol reagent (Invitrogen) according to the manufacturer’s instructions. The RNA samples were treated with RQ1 (DNAase) to eliminate any residual DNA and 1 μg thereof was used for reverse transcription using ReverTra Ace reverse transcriptase (Toyobo). Each cDNA was diluted to 200 ng/μl with dd H_2_O before use. Real-time qPCR was performed using the GoTaq® Green Master Mix kit (Promega) on a CFX96TM Real‐Time PCR Detection System apparatus (Bio‐Rad), and Nb18S was used as an internal control. All primers used in this study are listed in Supplementary Table 1.

### DNA extraction and PCR

Total DNA was extracted from leaves using the CTAB method as previously described (Akbergenov et al. 2006). PCR was performed using DNA polymerase (Takara) with AGV-specific primers (Supplementary Table 1).

### Immunoblot analysis

Immunoblot analysis was performed as previously described (Sun and Suzuki 2008). The polyclonal primary antibodies (1:3000) for the detection of TMV, CMV, and PVX were provided by Ying Wang (China Agriculture University). For the detection of ATG8 and ATG8-PE, total protein was extracted from systemic upper leaves and separated on a 15% SDS-PAGE gel with 6 mol/L urea and detected by a primary ATG8 antibody (1:3000) provided by Fei Yu (Northwest A&F University). Goat anti-rabbit immunoglobulin G-horseradish peroxidase (IgG-HRP; 1:5000; Proteintech) secondary antibody was used to detect the primary antibodies.

### Chemical treatment

For autophagy induction, BTH (100 µmol/L) was sprayed on *N. benthamiana* plants 24 and 48 h before sampling (Yang et al. 2018). For autophagy inhibition, *N. benthamiana* leaves were infiltrated with 10 mM 3-MA (Sigma-Aldrich) 8 h before sampling for DNA extraction (Huang et al. 2019). For the detection of TMV CP in BTH-treated plants, leaves infected with TMV were sprayed with BTH (100 µmol/L), and 72 h later, the leaves were sampled for immunoblotting analysis.

### MDC staining and fluorescence signal imaging

Plant tissues were selected and stained with 50 µmol/L MDC (Sigma-Aldrich) solution for 15 min in the dark. The tissues were washed twice with 10 mM PBS buffer and observed under a confocal microscope.

The fluorescence signals of GFP-ATG8f and MDC staining were observed using a confocal microscope (FC3000, Olympus) as previously described (Zhao et al. 2023). GFP was excited at 488 nm and detected at 510–550 nm. Chlorophyll autofluorescence was excited at 405 nm and detected at 635–708 nm. MDC was excited at 405 nm and detected at 430–460 nm (Contento et al. 2005).

### Bacterial disease assays

The *Pst* DC3000 inoculation and bacterial disease assay were conducted following previously established protocols (Yang et al. 2015). In brief, *Pst* DC3000 were cultured in LB medium at 28 °C, 24 h until reaching an OD_600_ of 1.0. The bacteria were then harvested by centrifugation, resuspended in infiltration buffer (10 mM MgCl_2_), and adjusted to OD_600_ = 0.002 before infiltration into plant leaves using a syringe. The infiltrated plants were maintained under high humidity (> 95%) to promote disease development for 2 days.

To quantify the *Pst* DC3000 bacterial populations, the plant leaves that had been infiltrated were surface-sterilized in 75% ethanol, rinsed with sterile water, and punched into leaf disks (1 square centimeter). These disks were ground in sterile water, subjected to serial dilutions, and plated on LB plates for colony-forming units (CFU) calculations. The experiments were repeated 9 times with 3 technical replicates for each biological trial.

### Fungal disease assays

The assays for *B. cinerea* desease were conducted as described previously (Xu et al. 2017). Briefly, the mycelia of *B. cinerea* were cultured on a PDA plate (9 cm) at room temperature for 5 days. The PDA blocks with *B. cinerea* myceria were cut from the edge of fungal colony by a sterile hole puncher (5 mm in a diameter) for inoculation. Plant leaves were placed with the fungal agar blocks and kept in high humidity (> 95%) to promote disease development. The disease lesions caused by the fungal inoculum were photographed and measured after 3 days of inoculation.

### 3,3′-diaminobenzidine (DAB) staining

Reactive oxygen species (ROS) activity was visualized using DAB as previously described previously (Yoon et al. 2009). In brief, *N. benthamiana* leaves were placed in a 9 cm plate with approximately 20 ml DAB solution (1 mg/ml). The plate was subjected to a vacuum for 15 min until the leaves were adequately infiltrated with the solution. Then the leaves were left to incubate overnight at room temperature. Subsequently, the stained leaves were transferred to 75% ethanol and boiled for a few minutes. Finally, the samples were rinsed twice with 50% ethanol before being photographed.

## Supplementary Information

Supplementary Material 1. Supplementary Table 1. A list of primers were used in this study.

Supplementary Material 2. Supplementary files 1. Original picture of CBB and western blot.

Supplementary Material 3. Figure S1. Inoculation of AGV on *N. benthamiana* plants. A. PCR detection of AGV DNA in upper systemic leaves of *N. benthamiana* plants. Primers specific for *N. benthamiana* 18S rRNA were used as the plant reference gene. B. An illustration depicting the experimental procedure for the pre-inoculation of AGV and inoculation of secondary heterologous viruses (PVX, CMV, and TMV).

## Data Availability

Data and material are available from corresponding author upon reasonable request.

## Abbreviations

AGV: Apple geminivirus 1
PVX: Potato virus X
CTV: Citrus tristeza virus
CMV: Cucumber moasic virus
TMV: Tobacco mosaic virus
RNA: Ribonucleic acid
PE: Lipid phosphatidylethanolamine
ATGs: Autophagy-related genes
PI3K: Phosphoinositide 3-kinase
VPS15: Vacuolar protein sorting 15
VPS34: Vacuolar protein sorting 34
DNA: Deoxyribonucleic acid
PCR: Polymerase chain reaction
RT-qPCR: Real-time reverse transcription quantitative PCR
qPCR: Real-time quantitative PCR
CTAB: Cetyltrimethylammonium Bromide
SDS-PAGE: Sodium dodecyl sulfate polyacrylamide gel electrophoresis
BTH: Benzothiadiazole
CP: Coat protein
MDC: Monodansylcadaverine
GFP: Green fluorescent proteins
LB: Lysogeny broth
CFU: Colony-forming units
PDA: Potato Dextrose Agar
ROS: Reactive oxygen species
DAB: 3,3′-Diaminobenzidine
dpi: Days post-inoculation
SA: Salicylic acid
JA: Jasmonic acid
ETH: Ethylene
PR: Pathogenesis-related protein
SAR: Systemic acquired resistance
MPK3: Mitogen-activated protein kinase 3
MYC2: Myelocytomatosis transcription factor 2
ERF3: Ethylene response factor 3
RNAi: RNA interference
DCL2: Dicer-2
DCL4: Dicer-4
AGO2: Argonaute-2
3-MA: 3-Methyladenine
TRV: Tobacco rattle virus
*Pst*DC3000: *P. syringae* Pv. tomato DC3000
siRNA: Small interfering RNAs
RISC: RNA-induced silencing complex
CLCuMuV: Cotton leaf curl Multan virus
TYLCV: Tomato yellow leaf curl virus
TYLCCNV: Tomato yellow leaf curl China virus
CBB: Coomassie brilliant blue
