# Supplementary material for: An asymptomatic geminivirus activates autophagy and enhances plant defenses against diverse pathogens
Source: Stress Biol. 2024 Oct 8;4(1):42. doi: 10.1007/s44154-024-00176-8 (PMC11461731; doi:10.1007/s44154-024-00176-8)
Supplement: Supplementary file 1 — Supplementary Material 1. Supplementary Table 1. A list of primers were used in this study. [file 44154_2024_176_MOESM1_ESM.docx]

Supplementary Table 1. A list of primers were used in this study.

| Primer Name | Oligonucleotide sequence (5’- 3’) |
| --- | --- |
| F-AGV-496 | CCTTCTGGACAAGAGCAA |
| R-AGV-496 | AGATGATTGGGTGAGTGT |
| F-Nb18S | GCAAGACCGAAACTCAAAGG |
| R-Nb18S | TGTTCATATGTCAAGGGCTGG |
| F-AGV-rt-121 | GTATTGCTTTGCCAGTCTC |
| R-AGV-rt-121 | ATCTCTCTCCCAAGGTGTT |
| F-NbATG5-rt | GAAGCTTATCTCCGAATCTCGTCTAAGC |
| R-NbATG5-rt | CCAACTTTCAACTGCAGGTGCATCTTG |
| F-NbATG7-rt | AGGTCTCGATGTCTAATCCTCTACGCCAG |
| R-NbATG7-rt | AATCAAATCAGACAAATGTCTGCAATCCTG |
| F-NbBecline1-rt | GACCTGCGTAAAGGAGTTTGCTGAC |
| R-NbBecline1-rt | CCAACAAACCAGTAGAGCACCCAC |
| F-NbATG1c-rt | TGGAAAGTCCCTCATCTGCACCTG |
| R-NbATG1c-rt | GCCTGCCTACCTCAACCTTCTCAT |
| F-NbATG4-rt | GGCGAAGCTGACTGGATACCTGTT |
| R-NbATG4-rt | ATCATCCTGCACGCCGACAATGTA |
| F-NbATG3-rt | GGAGGTGAGGAGGAGGAAGATATTCCAG |
| R-NbATG3-rt | CAGGCTCATGAGCCACAAGATATGC |
| F-NbVPS34-rt | GACATCTTCTGTCTGGCCTCC |
| R-NbVPS34-rt | CAGTTGCCAATACTCTGTATGGAG |
| F-NbVPS15-rt | GAGACCAAGGTGAAAGC |
| R-NbATG8f-rt | TGTTCAGGTCCCCGAATGTG |
| R-NbMPK3-rt | GTCGAAGGAGGATGGAACGG |
| R-NbERF3-rt | GATTCTCCGTCGGTAAAGG |
| F-NbDCL2-rt | GGAAGTAGCGGCTTTGTCAT |
| R-NbDCL2-rt | GCATCGTGGAATCTCAGGTAG |
| F-NbAGO2-rt | AAGGAGCATTTAGATAAGGATTCAG |
| R-NbAGO2-rt | TTCAGCCCGTACCATTTCACATA |
| F-NbDCL4-rt | TTTTATCCCAACAACTTCTACG |
| R-NbDCL4-rt | TCTTACGCAACCACTGATGACA |
| AGV1-F | tcatttcatttggagaggcctCTAAATGCTGGCCCTGCCCCC |
| AGV1-R | AAAATCCGCGGCCATCCGGTAATATTATACGGATGGCCGCC |
| AGV2-F | GGCGGCCATCCGTATAATATTACCGGATGGCC GCGGATTTT |
| AGV2-R | gccatgccgacccggggatccAGTACTTGGCGTTCCTCCC CCTGAAC |
| AGV3-F | CAGGGGGAGGAACGCCAAGTACTTGACGTTCCAGGAGAATGCG |
| AGV3-R | AAAATCCGCGGCCATCCGGTAATATTATACGG ATGGCCGCC |
| AGV4-R | gccatgccgacccggggatccagtACTTGGCGTTCCTCCCC CTGAAC |
