# Supplementary figures and images for: An asymptomatic geminivirus activates autophagy and enhances plant defenses against diverse pathogens
Source: Stress Biol. 2024 Oct 8;4(1):42. doi: 10.1007/s44154-024-00176-8 (PMC11461731; doi:10.1007/s44154-024-00176-8)

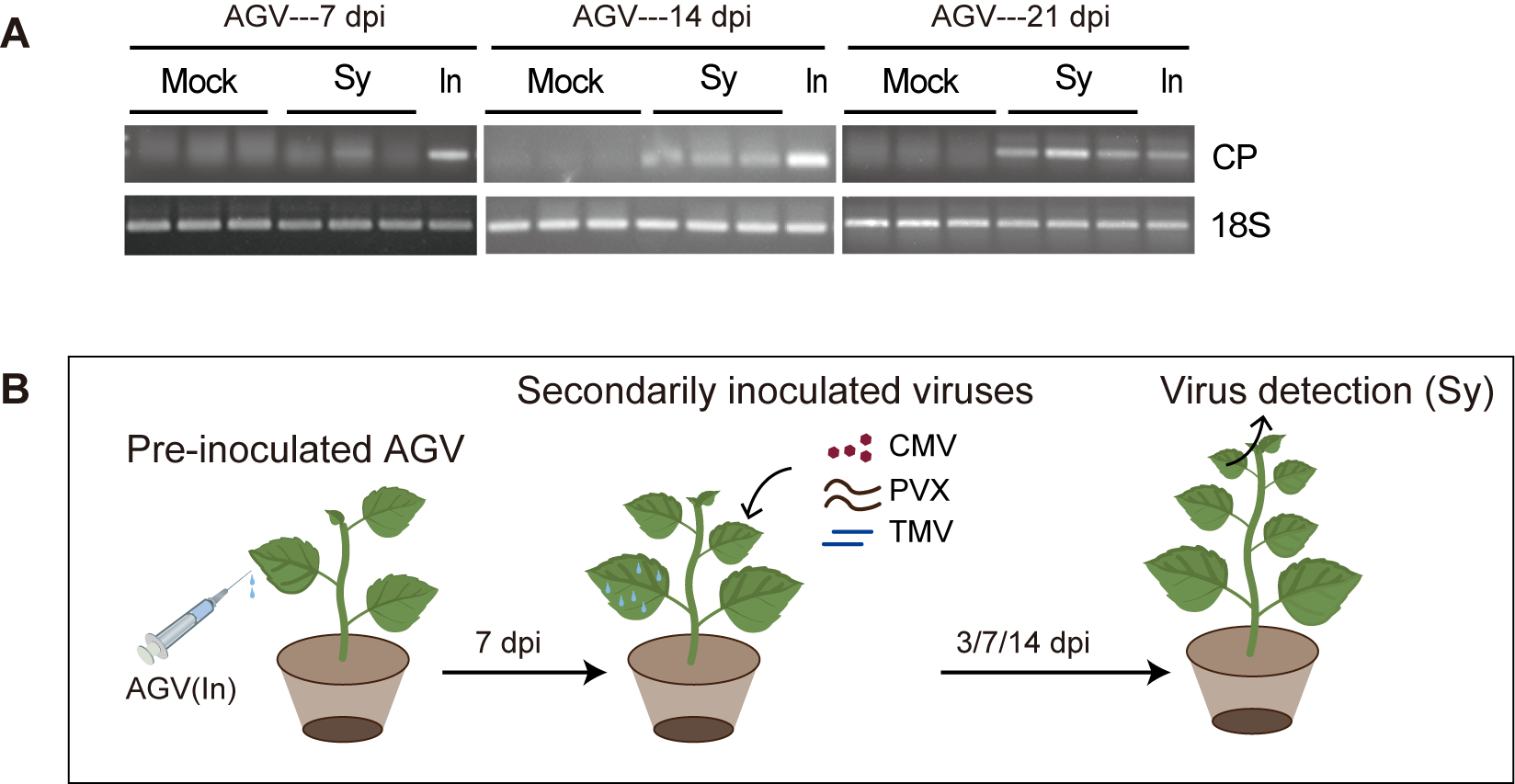

Supplement: Supplementary file 3 — Supplementary Material 3. Figure S1. Inoculation of AGV on N. benthamiana plants. A. PCR detection of AGV DNA in upper systemic leaves of N. benthamiana plants. Primers specific for N. benthamiana 18S rRNA were used as the plant reference gene. B. An illustration depicting the experimental procedure for the pre-inoculation of AGV and inoculation of secondary heterologous viruses (PVX, CMV, and TMV). [file 44154_2024_176_MOESM3_ESM.tif]
